# Self-assembling peptide hydrogel prevents esophageal stenosis after endoscopic submucosal dissection for esophageal squamous cell carcinoma: Multicenter prospective study

**DOI:** 10.1055/a-2816-5350

**Published:** 2026-03-25

**Authors:** Yuji Urabe, Yuichi Hiyama, Naoki Asayama, Yuzuru Tamaru, Yoji Sanomura, Shigeto Yoshida, Shinji Nagata, Yuko Hiraga, Ayako Takamori, Yasushi Orihashi, Toshio Kuwai, Shiro Oka

**Affiliations:** 1 68272 Department of Gastroenterology, Hiroshima University Hospital, Hiroshima, Japan; 2 13697 Gastroenterology, Hiroshima City North Medical Center, Asa Citizens Hospital, Hiroshima, Japan; 3 37086 Gastroenterology, Kure Medical Center and Chugoku Cancer Center, Kure, Japan; 4 37102 Department of Endoscopy, Hiroshima Prefectural Hospital, Hiroshima, Japan; 5 13697 Department of Endoscopy, Hiroshima City North Medical Center, Asa Citizens Hospital, Hiroshima, Japan; 6 68272 Clinical Research Center in Hiroshima, Hiroshima University Hospital, Hiroshima, Japan; 7 68272 Gastrointestinal Endoscopy and Medicine, Hiroshima University Hospital, Hiroshima, Japan; 8 68272 Department of Endoscopy, Hiroshima University Hospital, Hiroshima, Japan

**Keywords:** Endoscopy Upper GI Tract, Endoscopic resection (ESD, EMRc, ...), Benign strictures, Dilation, injection, stenting, Endoscopic ultrasonography, Esophageal cancer

## Abstract

**Background and study aims:**

Self-assembling peptide hydrogel (PuraStat) is a novel self-assembling peptide-based hemostatic agent that forms a transparent hydrogel upon contact with blood or bodily fluids. It has been reported to reduce the number of coagulations required with hemostatic forceps during gastrointestinal endoscopic procedures. In addition to its hemostatic effect, recent studies have suggested that PuraStat promotes wound healing. This study aimed to investigate the efficacy of PuraStat in preventing esophageal stenosis following endoscopic treatment of esophageal squamous cell carcinoma (ESCC).

**Patients and methods:**

This was a single-arm, multicenter, prospective study. Patients with esophageal squamous cell carcinoma who underwent endoscopic submucosal dissection and had a post-resection ulcer involving three-quarters or more of the full circumference of the esophagus received PuraStat application twice: once immediately after the procedure and once at second-look endoscopy 2 to 4 days later. The primary endpoint was incidence of esophageal stenosis within 12 weeks after endoscopic submucosal dissection. Secondary endpoints included the number of endoscopic balloon dilation procedures required and incidence of adverse events (AEs) and serious AEs.

**Results:**

Twenty patients were enrolled, all of whom received PuraStat application. Incidence of esophageal stenosis within 12 weeks was 20% (4/20). All cases of stenosis resolved with fewer than six endoscopic balloon dilation procedures and no patients developed refractory esophageal stenosis. AEs were observed in four patients; however, all resolved with conservative management.

**Conclusions:**

PuraStat application for post-endoscopic submucosal dissection ulcers in patients with ESCC may be useful for preventing esophageal stenosis.

## Introduction

Esophageal cancer remains the sixth leading cause of cancer-related mortality worldwide, with squamous cell carcinoma (SCC) being the predominant histological subtype in East Asian populations 1 . Endoscopic submucosal dissection (ESD) is a widely accepted minimally invasive curative treatment for esophageal SCC (ESCC) 2 3 4 5 6 . However, owing to the esophagus’s tubular anatomy, post-ESD esophageal stenosis (ES) risk increases with the extent of circumferential mucosal resection 7 . In particular, when the mucosal defect involves more than three-quarters of the esophageal circumference, ES incidence has been reported at 68% to 100% 7 8 9 10 .

Once ES develops, endoscopic balloon dilation (EBD) or bougie dilation is typically required to restore luminal patency. Some patients experience refractory ES, in which dysphagia persists despite multiple EBD sessions 11 . Consequently, even after curative resection, patients may continue to experience prolonged swallowing difficulties. EBD also carries risks of serious adverse events (AEs), such as perforation or bleeding, and frequent long-term EBD procedures impose a substantial economic burden 10 .

Safe and effective preventive therapies for esophageal stenosis are urgently needed, particularly in patients undergoing extensive or circumferential ESD. In Japan, steroid injection therapy has gained widespread use following reports of its efficacy in preventing post-ESD ES and is now considered the standard prophylactic treatment 11 12 . However, serious complications, including abscess formation and esophageal perforation, have been reported, indicating that safety is not fully established 13 14 .

Self-assembling peptide hydrogel (PuraStat) is a transparent, absorbable, topical hemostatic agent developed using self-assembling peptide technology. On contact with blood, it forms a gel-like structure that physically seals the bleeding site, achieving hemostasis. Because PuraStat is applied via a catheter rather than an injection needle, it carries a lower risk of perforation. Regarding ES prevention, an animal study in 11 pigs demonstrated that PuraStat reduced ES incidence and prevented esophageal lumen narrowing 15 16 . Based on these findings, we explored the safety and efficacy of PuraStat as a preventive treatment for post-ESD ES.

## Patients and methods

### Study design and participants

This study was approved by the Institutional Review Board of Hiroshima University Hospital (approval No. CRB2022–0014) on February 21, 2023. This single-arm, multicenter, prospective study was officially initiated on March 22, 2023. Twenty consecutive patients with ESCC undergoing ESD were prospectively enrolled. The trial commenced in March 2023 (first patient enrolled July 5, 2023) at Hiroshima University Hospital. Due to slow accrual, additional sites were added in April 2024, expanding enrollment to Hiroshima Prefectural Hospital, the National Hospital Organization Kure Medical Center and Chugoku Cancer Center, and Hiroshima City North Medical Center, Asa Citizens Hospital. Patients were eligible for first registration if they met all inclusion and no exclusion criteria.

This exploratory, proof-of-concept trial was not intended to statistically demonstrate efficacy or non-inferiority.

The main eligibility criterion was histologically confirmed superficial ESCC confined to the epithelium, lamina propria, or muscularis mucosa, without lymph node or distant metastasis. Lesions had to involve 50% or more of the esophageal circumference, with anticipated resection ranging from three-quarters to near-total circumference and longitudinal tumor length ≤ 50 mm. Patients were excluded if they had prior esophageal cancer treatment, radiation therapy to the cervicothoracic region, or previous esophageal or mediastinal surgery. Details of inclusion and exclusion criteria are described in the supplementary note.

Included patients underwent ESD. Those with post-ESD mucosal defects more than three-quarters of the circumference or nearly entire circumference were eligible for second registration, during which PuraStat was prophylactically applied to the post-ESD ulcer base to prevent ES. In addition to this prospective single-arm evaluation of PuraStat, an exploratory comparison was performed with a local steroid injection group derived from a retrospective observational cohort at our institution.

### Rationale for sample size

This exploratory trial aimed to obtain preliminary data on PuraStat potential effectiveness and safety for preventing post-ESD ES. The sample size was set at 20 patients based on feasibility and proof-of-concept requirements. No formal hypothesis-testing or sample-size calculation was performed because the effect size of PuraStat was unknown and the study was not powered for statistical efficacy.

### Protocol, treatment, and follow-up procedures

Methods of PuraStat application.Video 1

**Fig. 1:**
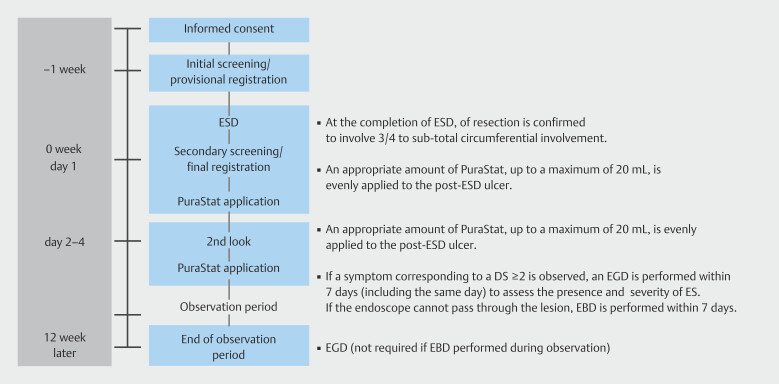
Outline of the study protocol for the PuraStat application group.

The study flow is summarized in Fig. 1 . Immediately after ESD, PuraStat was evenly applied over the entire post-ESD ulcer. Because no established dosing criteria exist for stricture prevention, the volume of PuraStat was determined in accordance with the Japanese package insert, which states that the maximum volume per endoscopic procedure should not exceed 20 mL; therefore, 20 mL was set as the upper limit. In this study, PuraStat was applied twice: immediately after ESD and again during second-look endoscopy performed 2 to 4 days later, with each application limited to a maximum of 20 mL. Application continued until the ulcer was fully covered. Between days 2 to 4, follow-up endoscopy was performed to confirm hemostasis, after which PuraStat was reapplied evenly over the ulcer base (maximum 20 mL) ( Video 1 ). If the second application could not be performed by day 4, it was omitted.

Patients were followed for 12 weeks to evaluate ES development. Dysphagia score (DS) was assessed every 2 weeks. If DS ≥ 2 was reported, endoscopy was performed within 7 days to assess stenosis. The ability of a standard 9.6- to 10.4-mm endoscope to pass through the esophagus was evaluated. EBD was performed if passage failed. Combination therapy with EBD, bougie dilation, or local steroid injections was not permitted. EBD was withheld if DS ≥ 2 but the 9.6- to 10.4-mm endoscope could pass. EBD used balloons of 15- to 18-mm diameter.

At 12 weeks post-ESD, esophagogastroduodenoscopy with a 9.6- to 10.4-mm endoscope confirmed passage through the treated area. DS evaluation was allowed within ± 7 days of the scheduled assessment point, and the 12-week endoscopy was permitted within a ± 14-day window.

### Endpoints and assessments

The primary endpoint was the proportion of patients developing ES within 12 weeks. Secondary endpoints included stenosis-free survival, number of EBDs, proportion of patients with DS ≤ 1 at 12 weeks, and AE incidence. ES was defined as DS ≥ 2 plus inability to pass a 9.6- to 10.4-mm endoscope. Endoscopy was required within 7 days of DS ≥ 2. AEs were graded per Common Terminology Criteria for Adverse Events, version 4.0.

### Comparative analysis with previously reported stenosis prevention methods

To evaluate PuraStat efficacy, outcomes were compared with historical data from patients receiving local triamcinolone injection monotherapy applied directly to the post-ESD ulcer base, without adjunctive therapies. This cohort included 78 lesions in 74 patients treated at Hiroshima University Hospital between November 2015 and July 2023. All patients had tumors involving half to less than the full esophageal circumference, with post-ESD defects more than three-quarters to subtotal circumferential, and no history of radiation therapy or esophageal/mediastinal surgery.

Baseline differences were adjusted using 1:1 propensity score matching (PSM) with logistic regression based on age, sex, tumor location, tumor length, and circumferential extent. Nearest-neighbor matching without replacement and a caliper of 0.2 standard deviations of the logit were used. Covariate balance was assessed using standardized mean differences.

### Ethics statement

This study was conducted in accordance with the Declaration of Helsinki and was approved as a clinical trial by the Certified Review Board of Hiroshima University (approval no. CRB2022–0014). Written informed consent was obtained from all participants after thoroughly explaining the study protocol, including the potential risks and benefits. For patients receiving triamcinolone, the study protocol was disclosed via opt-out due to retrospective data use.

### Statistical analyses

Quantitative variables are expressed as means ± standard deviations or percentages. Categorical variables were analyzed using chi-squared test with Yates’s correction or Fisher’s exact test. Continuous variables were assessed using Student’s t-test or Mann-Whitney U test. Statistical significance was set at *P* < 0.05.

## Results

### Clinicopathological characteristics of enrolled cases

At the first registration, a total of 22 patients were enrolled. Two patients were excluded because the post-ESD mucosal defect involved less than three-quarters of the esophageal circumference. Ultimately, 20 patients were included in the second registration and received prophylactic PuraStat to prevent ES.

Seventeen patients were enrolled from Hiroshima University Hospital, two from Hiroshima City North Medical Center, Asa Citizens Hospital, and one from the National Hospital Organization Kure Medical Center and Chugoku Cancer Center. Clinicopathological characteristics of the 20 patients who received PuraStat are summarized in Table 1 .

**Table:** **Table 1** Clinical characteristics of ESCC cases in which PuraStat was used for stricture prevention.

Variables	n = 20
Age, years, mean ± SD	66 ± 13
Sex, male, n (%)	18 (90)
Tumor location	
Ut, n (%)	00 (0)
Mt, n (%)	18 (90)
Lt, n (%)	2 (10)
Tumor size, mm	35 ± 8
Circumferential extent of the tumor	
1/2–2/3	14 (70)
2/3–3/4	04 (20)
3/4-subtotal	02 (10)
Post-resection ulcer circumferential extent	
3/4–5/6	15 (75)
5/6–7/8	03 (15)
7/8-subtotal	2 (10)
Resected specimen length, mm	46 ± 10
PuraStat volume at first application (mL)	12 ± 5
PuraStat volume at second application (mL)*	9 ± 4
ESCC, esophageal squamous cell carcinoma; Mt, middle thoracic; Lt, lower thoracic; SD, standard deviation; Ut, upper thoracic. *In one case, perforation occurred during ESD, and to avoid potential adverse effects at the perforation site, follow-up endoscopy and the second application of PuraStat were not performed.	

### ES prevention outcomes with PuraStat

Results of the primary and secondary endpoints are presented in Table 2 . The mean volume of PuraStat applied at the primary enrolling institution was 13.7 ± 3.6 mL for the first application and 10.2 ± 3.8 mL for the second, whereas the corresponding volumes at other institutions were 4.2 ± 0.8 mL and 4.3 ± 0.9 mL, respectively.

**Table:** **Table 2** Outcomes of esophageal stricture prevention using PuraStat.

Variables	n = 20
Primary endpoint	
12-week stenosis rate	20 (4/20)
Secondary endpoints	
Stricture-free survival (month, range)	73 ± 22 (14–84)
Mean no. of dilations in stenosis cases (range)	2.2 (1–5)
Rate of dysphagia score ≥ 2 at 12 weeks (%)	5 (1/20)
Incidence of adverse events (%)	20 (4/20)
Incidence of serious adverse events (%)	20 (4/20)
Postoperative bleeding	2
Perforation	1
Pneumonia	1

Incidence of ES within 12 weeks after ESD, which was the primary endpoint, was 20% (4 of 20 patients). Mean stenosis-free survival time, a secondary endpoint, was 73.2 days, with the earliest occurrence of ES observed at 14 days post-treatment. Among the four patients who developed ES, the mean number of EBD sessions required for resolution was 2.2 (range: 1–5). At 12 weeks post-ESD, 95% of patients (19/20) had a DS ≤ 1 and only one patient had a DS of 2.

Incidence of AEs and serious AEs was 20% (4/20) each. Regarding serious AEs specifically, two patients experienced post-procedural bleeding, one experienced perforation, and one developed pneumonia. Of the two patients with bleeding, one was diagnosed with hemophilia B during follow-up and the other had underlying liver cirrhosis and was taking low-dose aspirin. The perforation occurred during the ESD procedure and was successfully managed conservatively. The pneumonia occurred in a patient who developed ES, suggesting possible aspiration pneumonia secondary to stenosis.

Details about the four patients who developed ES are shown in Table 3 . Of these, one patient experienced esophageal perforation during ESD. Two patients had tumors involving three-quarters or more of the esophageal circumference and post-ESD mucosal defects extending seven-eighths of the circumference. Only two patients in this study had post-ESD mucosal defects extending to seven-eighths or more of the esophageal circumference, and both developed esophageal stenosis.

**Table:** **Table 3** Detailed clinical backgrounds of patients who developed esophageal stenosis.

**No.**	**Age, years**	**Sex**	**location**	**Tumor size (mm)**	**Circumferential extent of the tumor**	**Circumferential extent of the resected ulcer**	**Resected size (mm)**	**Muscularis propria injury**	**PuraStat volume**		**Stenosis-free period (days)**	**Number of EBDs**	**Adverse events**
									**1st (mL)**	**2nd (mL)**			
1	67	Male	Mt	30	1/2–2/3	3/4–5/6	40	yes	10	0	34	5	Perforation
2	42	Male	Mt	20	1/2–2/3	5/6–7/8	40	-	15	15	14	1	-
3	73	Male	Mt	42	3/4-subtotal	7/8-subtotal	50	-	5	5	40	4	-
4	81	Male	Mt	40	3/4-subtotal	7/8-subtotal	50	-	15	12	43	3	Pneumonia
EBD, endoscopic balloon dilation; Mt, middle thoracic.													

**Fig. 2:**
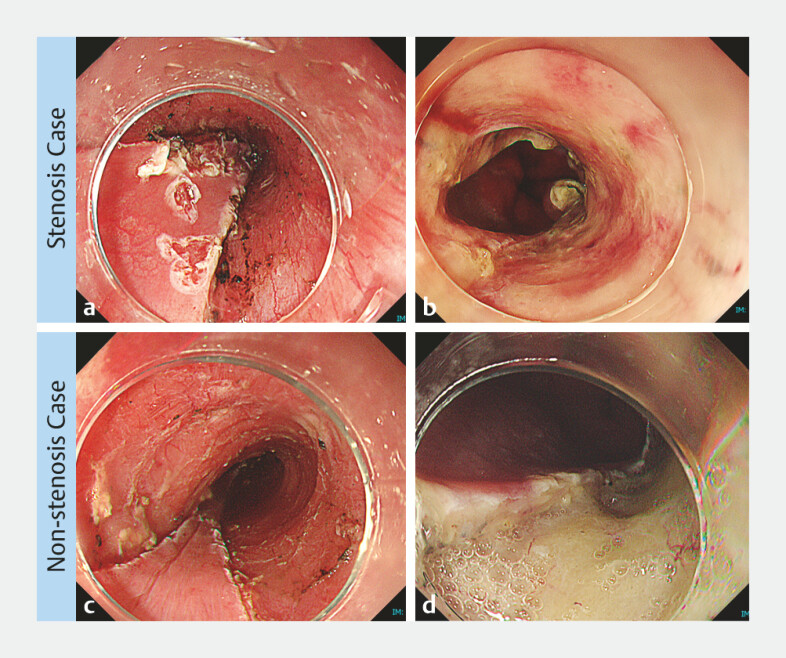
Representative endoscopic images of the ulcer base immediately after ESD and at second-look endoscopy in patients who developed or did not develop esophageal stenosis. **a,b** Representative case with esophageal stenosis. **a** Endoscopic image immediately after ESD. **b** Endoscopic image at second-look endoscopy, showing no visible residual PuraStat on the ulcer base within the visually accessible area. **c,d** Representative case without esophageal stenosis. **c** Endoscopic image immediately after ESD. **d** Endoscopic image at second-look endoscopy, showing visible residual PuraStat on the ulcer base.

Representative endoscopic images of the ulcer base at second-look endoscopy from a patient who developed esophageal stenosis and one who did not are shown in Fig. 2 . In the patient who developed stenosis, no visible residual PuraStat was observed on the ulcer base within the visually accessible area at second-look endoscopy. In contrast, in the patient who did not develop stenosis, residual PuraStat was clearly visible on the ulcer base at second-look endoscopy.

### Comparison of ES prevention outcomes between PuraStat and triamcinolone injection

The primary outcome was compared between the patients who received PuraStat and those who received prophylactic local triamcinolone injections after ESD. Incidence of ES within 12 weeks in the triamcinolone group was 20% (15/52), which was comparable to the 20% (4/20) incidence observed in the PuraStat group, indicating similar treatment outcomes ( Table 4 ).

**Table:** **Table 4** Comparison of stricture prevention using PuraStat vs. triamcinolone.

**Variable**	**PuraStat**	**Control**	***P* value **
	**n = 20**	**n = 77**	
Age, years (mean ± SD)	66 ± 13	68 ± 9	0.49
Sex, male, n (%)	18 (90)	70 (91)	1
Tumor location			0.044
Ut	0 (0)	14 (18)	
Mt	18 (90)	48 (62)	
Lt	2 (10)	15 (20)	
Tumor size, mm (mean ± SD)	35 ± 8	34 ± 9	0.67
Circumferential extent of the tumor			0.89
1/2–2/3	14 (70)	56 (73)	
2/3–3/4	4 (20)	12 (16)	
3/4-subtotal	2 (10)	9 (11)	
Resected specimen length, mm (mean ± SD)	46 ± 10	46 ± 11	0.8
Post-resection ulcer circumferential extent			0.71
3/4–5/6	15 (75)	56 (73)	
5/6–7/8	3 (15)	12 (16)	
7/8-subtotal	2 (10)	9 (11)	
Presence of muscularis propria injury	1 (5)		1
Tumor depth			0.4
EP	4 (20)	11 (14)	
LPM	13 (65)	40 (52)	
MM	1 (5)	18 (23)	
SM1	1 (5)	2 (3)	
SM2	1 (5)	6 (8)	
12-week stenosis rate	4 (20)	15 (19)	1
EP, epithelial; LPM, lamina propria mucosa; Lt, lower thoracic; MM, muscularis mucosa; Mt, middle thoracic; SD, standard deviation; SM, superficial submucosa; SM2, deep submucosa; Ut, upper thoracic.			

Clinicopathological factors were compared between the two groups, and no significant differences were found in age, sex, tumor length, circumferential extent, resection diameter, muscularis propria injury, or pathological depth of invasion. However, tumor location differed significantly between the groups. After PSM, incidence of ES within 12 weeks was 15% (3/20) in the triamcinolone group, which was not significantly different from that in the PuraStat group ( Table 5 ).

**Table:** **Table 5** Comparison of stricture prevention using PuraStat vs. triamcinolone after propensity score matching.

**Variable**	**PuraStat**	**Control**	***P* value **
	**n = 20**	**n = 20**	
Age, years (mean ± SD)	66 ± 13	70 ± 8	0.27
Sex, male, n (%)	18 (90)	18 (90)	1
Tumor location			0.083
Ut	0 (0)	1 (5)	
Mt	18 (90)	12 (60)	
Lt	2 (10)	7 (35)	
Tumor size, mm (mean ± SD)	35 ± 8	38 ± 8	0.26
Circumferential extent of the tumor			0.92
1/2–2/3	14 (70)	15 (75)	
2/3–3/4	4 (20)	3 (15)	
3/4-sub total	2 (10)	2 (10)	
Resected specimen length, mm (mean ± SD)	46 ± 10	48 ± 10	0.53
Post-resection ulcer circumferential extent			1
3/4–5/6	16 (80)	15 (75)	
5/6-sub total	2 (10)	3 (15)	
5/6-sub total	2 (10)	2 (10)	
Presence of muscularis propria injury	1 (5)	2 (10)	1
Tumor depth			0.98
EP	4 (20)	3 (15)	
LPM	13 (65)	13 (65)	
MM	1 (5)	2 (10)	
SM1	1 (5)	1 (5)	
SM2	1 (5)	1 (5)	
12-week stenosis rate	4 (20)	3 (15)	1
EP, epithelial; LPM, lamina propria mucosa; Lt, lower thoracic; MM, muscularis mucosa; Mt, middle thoracic; SD, standard deviation; SM, superficial submucosa; SM2, deep submucosa; Ut, upper thoracic.			

## Discussion

In animal experiments involving circumferential esophageal mucosal resections in pigs, matrix-assisted laser desorption/ionization imaging mass spectrometry confirmed presence of PuraStat 24 hours after application, suggesting that it may affect the ulcer base over an extended period 16 . Furthermore, in these animal studies, the PuraStat-treated group showed thicker inflammatory cell infiltrates, higher collagen I/III ratios, and significantly longer neoepithelial regeneration compared with the untreated group 16 . These findings suggest that PuraStat promotes reepithelialization. PuraStat has been reported to facilitate wound healing in humans. Uraoka et al. 17 demonstrated that PuraStat application after gastric ESD promoted mucosal regeneration and ulcer healing. Based on these findings, we hypothesized that PuraStat could be effective for preventing post-ESD ES following endoscopic esophageal cancer resection and we conducted this study accordingly.

In this study, ES occurred in four of 20 patients (20%) who underwent PuraStat-based stenosis prevention following ESDs for ESCC lesions measuring ≤ 5 cm in length with circumferential extension from half to subtotal. Moreover, esophageal stenosis developed in all patients with post-ESD mucosal defects extending to seven-eighths or more of the esophageal circumference. Even among patients with mucosal defects involving more than three-quarters of the circumference, a greater circumferential extent has been reported to be associated with a higher stenosis risk, even when a prophylactic triamcinolone injection is used. Nagami et al. 18 reported that defects involving more than five-sixths of the circumference pose a high ES risk, whereas Chen et al. 19 identified defects involving seven-eighths or more as a high-risk factor. The present findings suggest that PuraStat-based ES prevention follows a similar trend. These findings suggest that PuraStat alone may be insufficient to prevent stenosis with extremely extensive circumferential mucosal defects. For such high-risk lesions, combination strategies incorporating additional prophylactic measures may be necessary. One potential approach is combination therapy with local steroid administration. Both PuraStat and steroids exert anti-inflammatory and antifibrotic effects, and their combined use may enhance these biological actions. In addition, PuraStat may provide a scaffold that facilitates epithelial regeneration through its wound-healing properties, potentially complementing the effects of steroids. Further basic and clinical studies are warranted to evaluate the efficacy and safety of such combination strategies and to establish optimal preventive approaches for esophageal stenosis in patients with extensive post-ESD mucosal defects.

In addition, one stenosis case was observed, despite a mucosal defect involving less than five-sixths of the circumference. The patient experienced esophageal perforation during ESD and did not undergo a second PuraStat application. Although absence of a second application may have contributed to stenosis development, the perforation itself was considered to have had a greater impact. Indeed, muscular layer injury has been reported as a risk factor for ES, even under steroid-based prophylaxis 20 21 , and the present case suggests that such an injury may also be a risk factor when using PuraStat for stenosis prevention. We also compared

PuraStat preventive effects on ES with those of prophylactic triamcinolone injections. ES incidence after triamcinolone injection ranges from 10% to 19% 11 12 17 22 . However, these studies differed from ours in terms of eligibility criteria and had relatively small sample sizes. Therefore, to minimize these differences, we performed a comparative analysis using cases from Hiroshima University, which had the largest number of registered patients and aligned with the inclusion criteria as closely as possible. In the group that received prophylactic triamcinolone injections, ES incidence was 15%, which was not significantly different from the 20% incidence observed in the group that received PuraStat. Although a 5% difference was observed, the stenosis rate before matching was 20%, suggesting that this difference may be attributable to the influence of the sample size. Furthermore, previous reports have shown that, without any prophylactic intervention, post-ESD ES incidence ranges from 30% to 75% 11 12 22 . Based on these findings, our results suggest that PuraStat’s preventive effect is comparable to that of triamcinolone. However, this study was exploratory in nature, no direct comparison was made between PuraStat, triamcinolone, and it lacked a no-treatment group. Therefore, a randomized controlled trial (RCT) is warranted to validate the efficacy of PuraStat in preventing ES after ESDs.

In this study, delayed bleeding was observed as an AE in two patients. Both patients were on anticoagulant medications or had underlying bleeding diatheses. PuraStat is a hemostatic agent designed to reduce the number of coagulation attempts using hemostatic forceps in cases of oozing-type bleeding and previous reports have suggested its effectiveness in preventing delayed bleeding as well 23 24 25 . However, a recent large-scale RCT reported no significant preventive effects of PuraStat on delayed bleeding after colorectal endoscopic mucosal resections 26 . Given these findings, when PuraStat is not directly applied to the bleeding site, as in this study, it may be necessary to perform sufficient hemostatic procedures, such as coagulation with forceps, before applying PuraStat to ensure adequate bleeding control.

In this study, a maximum volume of 20 mL per application was used for PuraStat. At the time of study planning, the optimal volume required to effectively prevent post-ESD esophageal stricture had not been established; therefore, the domestic regulatory limit of 20 mL per procedure was adopted as the upper limit. This approach allowed feasibility and appropriate use of PuraStat to be evaluated safely within the maximum dose permitted in clinical practice. In the present study, differences in PuraStat volumes among institutions were observed. These differences may reflect the exploratory nature of the study, which was designed to evaluate safety and potential efficacy of PuraStat. In this early-phase setting, a cautious application strategy aimed at maximizing the preventive effect may have been adopted, contributing to the observed variation in PuraStat usage. Future studies should systematically investigate an optimal dosing strategy to prevent post-ESD esophageal stenosis effectively while avoiding unnecessary overuse.

Recently, Yang et al. 27 reported that PuraStat is effective in preventing post-ESD ES. Our study differs in several important aspects. First, their trial was conducted in the United States, whereas our study was performed in Japan and exclusively included Japanese patients. Genetic background, clinical characteristics, and endoscopic practice may differ in Japanese patients. Second, their study included Barrett’s esophageal adenocarcinoma cases, whereas our study focused solely on superficial ESCC, the predominant histological esophageal cancer type in Japan. By targeting this population, our study provides unique and clinically meaningful insights into PuraStat’s role in preventing post-ESD strictures in patients with ESCC.

Although PuraStat demonstrated a stricture-preventive effect comparable to that of local steroid injection, its higher cost remains an important consideration. In this study, PuraStat was applied twice, resulting in a larger total volume than typically used for routine hemostasis, which may raise concerns regarding cost-effectiveness compared with steroid-based therapy. At present, steroid-based therapy remains the most established prophylactic approach for preventing post-ESD esophageal stenosis. Therefore, in patients who are intolerant of steroids or for whom steroid use is contraindicated, no alternative prophylactic strategy is currently established. In such cases, PuraStat may be a reasonable therapeutic option despite its higher cost. Nevertheless, optimization of PuraStat use is warranted. Previous studies have suggested that single-session PuraStat application may be effective, indicating that refinement of dosing volume and the number of applications could improve cost-effectiveness 27 . Future studies should aim to establish an optimal dosing strategy that balances efficacy, safety, and economic considerations.

This study had some limitations. First, the sample size was small and it was a single-arm trial. However, because this was an exploratory study, we consider it a necessary step toward the next phase of investigation. Second, PuraStat was administered twice to each patient. At the time of study design, it was unclear whether a sufficient amount of PuraStat could be applied to the ulcer base immediately after endoscopic treatment; therefore, we adopted a protocol involving two separate applications. Therefore, it remains unclear whether a single application of PuraStat immediately after esophageal ESD is sufficient to prevent stenosis. However, Yang et al. 27 reported that a single application of PuraStat immediately after ESD effectively prevented ES. This finding suggests that a single application during ESD may be sufficient to prevent stenosis.

Third, in this study, the degree of residual PuraStat at second-look endoscopy was not predefined as an evaluation item in the study protocol. In addition, second-look endoscopy was scheduled between 2 and 4 days after ESD, resulting in variability in observation timing. Therefore, quantitative or statistical assessment of PuraStat persistence was not feasible. Future studies should incorporate a standardized evaluation of gel persistence, ideally by assessing the degree of residual gel on the day after ESD, to clarify the relationship between gel persistence and stricture prevention. These observations were descriptive and based on representative cases, as systematic assessment was performed all patients.

## Conclusions

This is the first exploratory study to evaluate the efficacy of PuraStat specifically for prevention of post-ESD ES in patients with ESCC. Based on the results, incidence of ES was 20% when PuraStat was applied after ESD for ESCC lesions measuring ≤ 5 cm in length with extension from half to sub-total circumferential. This incidence may be comparable to that reported for local triamcinolone injection therapy. Importantly, none of the patients required six or more EBD sessions, indicating that no patient developed refractory stenosis. Although AEs were observed, all resolved with conservative management. These findings suggest that applying PuraStat to the post-ESD ulcer base may be effective in preventing ES. To validate these findings further, a prospective RCT comparing PuraStat with steroid-based prophylaxis or no prophylactic intervention is warranted.

## Supplementary Material

Supplementary Material Zusatzmaterial
