# Supplementary material for: Self-assembling peptide hydrogel prevents esophageal stenosis after endoscopic submucosal dissection for esophageal squamous cell carcinoma: Multicenter prospective study
Source: Endosc Int Open. 2026 Mar 25;14:a28165350. doi: 10.1055/a-2816-5350 (PMC13062683; doi:10.1055/a-2816-5350)
Supplement: Supplementary file 2 — Supplementary Material [file 10-1055-a-2816-5350_28194564.pdf]

[Heading 1]Inclusion and exclusion criteria for eligible patients

Eligibility required histologically confirmed primary squamous cell carcinoma, including high-grade intraepithelial neoplasia or basaloid carcinoma, based on endoscopic biopsy. The tumor had to be localized to the thoracic esophagus, with biopsy findings indicating invasion limited to the epithelium, lamina propria, or muscularis mucosa. There had to be no evidence of lymph node or distant metastases on contrast-enhanced computed tomography (CT) from the neck to the abdomen or on positron emission tomography-CT. For patients with renal dysfunction, contrast agent allergies, or bronchial asthma, non-contrast CT findings were acceptable. Patients were required to have a tumor involving at least half but not the entire circumference of the esophagus, with an expected resection extent ranging from three-quarters to near-complete circumferential involvement. The tumor length along the longitudinal axis had to be ≤ 50 mm (assessed endoscopically). In cases of multiple lesions, all non-primary lesions—defined as those other than the one with the widest circumferential involvement—had to involve less than half of the esophageal circumference.

Eligible patients were aged between 18 and 85 years at the time of informed consent and had an Eastern Cooperative Oncology Group performance status of 0 or 1. Patients were excluded if they had a history of radiation therapy to the cervicothoracic region, lungs, or mediastinum, or if they had previously undergone esophageal or mediastinal surgery, including procedures for benign conditions. A history of prior treatment for esophageal cancer also led to exclusion. All participants were required to have a dysphagia score of 0 at the time of enrollment. Laboratory values obtained within 90 days before provisional registration had to meet the following criteria: white blood cell count between 2,000/mm^3 and 12,000/mm^3;

hemoglobin level ≥ 8.0 g/dL; platelet count ≥ 100,000/mm³; total bilirubin level ≤ 2.0 mg/dL; aspartate aminotransferase and alanine aminotransferase levels ≤ 150 IU/L; serum creatinine level ≤ 2.0 mg/dL; and hemoglobin A1c (NGSP) < 7.0%. Written informed consent was obtained from all participants before enrollment.

Patients were excluded if they had active concomitant cancer requiring treatment, an active infection requiring systemic therapy, or fever ≥ 38°C at the time of enrollment. Additional exclusion criteria included pregnancy, potential pregnancy, being within 28 days postpartum, or breastfeeding. Psychiatric disorders or symptoms that could interfere with study participation were also grounds for exclusion. Patients receiving ongoing systemic corticosteroid therapy, other immunosuppressants, or inhaled corticosteroid-containing medications were excluded. Other exclusionary conditions were uncontrolled hypertension, unstable angina that developed or worsened within the preceding 3 weeks, myocardial infarction within the past 6 months, and respiratory disease requiring continuous oxygen therapy. A history of any surgery within the previous 3 months or known hypersensitivity to peptide- or protein-based products also led to exclusion.
